# Comparison of Liver Biomarkers in 288 COVID-19 Patients: A Mono-Centric Study in the Early Phase of Pandemic

**DOI:** 10.3389/fmed.2020.584888

**Published:** 2021-01-15

**Authors:** Haozhi Fan, Jinyuan Cai, Anran Tian, Yuwen Li, Hui Yuan, Zhengyi Jiang, Yunxi Yu, Lili Ruan, Pingping Hu, Ming Yue, Nian Chen, Jun Li, Chuanlong Zhu

**Affiliations:** ^1^Department of Information, The First Affiliated Hospital of Nanjing Medical University, Nanjing, China; ^2^Department of Infectious Disease, The First Affiliated Hospital of Nanjing Medical University, Nanjing, China; ^3^Department of Pediatrics, The First Affiliated Hospital of Nanjing Medical University, Nanjing, China; ^4^State Key Laboratory for Diagnosis and Treatment of Infectious Diseases, Collaborative Innovation Center for Diagnosis and Treatment of Infectious Diseases, College of Medicine, The First Affiliated Hospital, Zhejiang University, Hangzhou, China; ^5^Emergency Department, Huangshi Hospital of Traditional Chinese Medicine, Huangshi, China; ^6^Department of Anesthesiology, The Fifth Hospital of Huangshi, Huangshi, China; ^7^Department of Tropical Diseases, The Second Affiliated Hospital of Hainan Medical University, Haikou, China

**Keywords:** COVID-19, SARS-CoV-2, liver biomarkers, liver injury, hepatic dysfunction

## Abstract

**Background and Aims:** Recent reports have indicated that hepatic dysfunction occurred in a proportion of patients with coronavirus disease 2019 (COVID-19). We aimed to compare and describe the liver biomarkers in different subtypes of COVID-19 patients.

**Methods:** This study enrolled 288 COVID-19 patients in Huangshi Hospital of Traditional Chinese Medicine. All patients were divided into ordinary, severe, and critical groups according to the *Diagnosis and Treatment Protocol for Novel Coronavirus Pneumonia (Trial Version 7)*. Demographic, clinical characteristics and liver biomarkers were compared among the three groups.

**Results:** During hospitalization, AST, TBiL, and ALP levels in ordinary and severe patients fluctuated within the normal range with a rising trend in critical patients except AST. ALT and GGT levels fluctuated within the normal range showing an upward trend, while LDH levels in the critical group exceeded the normal range. Prealbumin showed an upward trend, especially in the severe group. At discharge, AST and LDH levels in ordinary and severe groups were lower than their baselines but increased in the critical group. In contrast to albumin, TBiL levels were increased in ordinary and critical groups while decreased in the severe group. The stratified analysis revealed factors affecting liver function in critical cases included highest temperature ≥38.0°C, age ≥60 and symptom of hypoxemia.

**Conclusions:** COVID-19 can cause severe hepatic dysfunction in critical patients, requiring early monitoring and intervention. LDH, ALP, GGT, TBiL, prealbumin, and albumin may be helpful for evaluating and predicting disease prognosis due to their correlation with disease severity in COVID-19.

## Introduction

Since December 2019, a pneumonia of unknown cause broke out in Wuhan. Epidemiological evidence shows that this pneumonia can spread among people through close contact and respiratory droplets, and people are generally susceptible (1–3). Different from severe acute respiratory syndrome (SARS) and middle east respiratory syndrome coronavirus (MERS) (4), a novel coronavirus named severe acute respiratory syndrome coronavirus 2 (SARS-CoV-2) was identified as the pathogen. Subsequently, this unique pneumonia was named coronavirus disease 2019 (COVID-19) by World Health Organization (WHO). Common clinical manifestations of SARS-CoV-2 infection include fever, fatigue and dry cough (5–9). In the early stage of infection, chest computed tomography (CT) only exhibits multiple small spot shadows and interstitial changes, and this then develops into multiple ground-glass opacities and infiltration in both lungs. In some severe cases, acute respiratory distress syndrome (ARDS), sepsis and even multiple organ failure may occur (5). Although the fatality rate of SARS-CoV-2 is not as high as that of SARS, its transmission and pathogenicity are even stronger. COVID-19 has already become a worldwide pandemic, so it is urgent to control the epidemic.

At present, there is still no specific drug or vaccine for COVID-19. Patients mainly receive symptomatic and supportive treatment during hospitalization to prevent serious complications. Clinical reports show that ALT, AST, TBiL, and other liver-related biochemical indexes of some patients with COVID-19 have increased to varying degrees (5–9). It suggests that in addition to cardiopulmonary injury, substantial hepatic impairment also exists, especially in severe and critical cases. Unfortunately, there is little research involving the mechanism of liver injury caused by COVID-19, nor does any pathological report prove that SARS-CoV-2 can directly attack the liver. Thus, the cause of liver dysfunction in COVID-19 remains unclear.

In clinical operations, serum biochemical examinations are more accessible than complicated operations such as liver biopsy and ultrasonography. Thus, indicators related to liver function in blood biochemical examination were selected as observation indicators. In serum biochemical examination, AST, ALT, and LDH are indicators of liver function that reflect the damage and severity of liver cells. ALP, GGT, and TBiL are indicators of liver function that reflect bilirubin metabolism and cholestasis. As for prealbumin and albumin, they are indicators that reflect liver synthesis and reserve function. In this study, we compared the clinical manifestations and liver biomarkers among different subtypes of COVID-19 patients, focusing on the baseline characteristics and dynamic change trend of the above liver biomarkers at admission and during hospitalization. Besides, we discussed the potential mechanism of hepatic impairment in critical cases to provide novel insights for clinical decision-making and drug development.

## Methods

### Study Participants

This retrospective study enrolled a total of 288 COVID-19 patients in Huangshi Hospital of Traditional Chinese Medicine in Hubei Province from January to April 2020. Among them, male and female patients numbered 147 and 141, respectively. According to the *Diagnosis and Treatment Protocol for Novel Coronavirus Pneumonia (Trial Version 7)* (10), COVID-19 patients were confirmed by positive real-time reverse transcriptase-polymerase chain reaction (RT-PCR) and chest CT test. Clinical and laboratory information of these patients should be completed.

Patients infected with other common respiratory viruses, including influenza A and B viruses, respiratory syncytial virus, parainfluenza virus, adenovirus, SARS coronavirus, or MERS coronavirus or a combination with chronic liver diseases, such as viral hepatitis, autoimmune liver disease, alcoholic fatty liver disease, or liver cancer, were excluded from this study.

### Data Collection

Clinical information, including age, gender, epidemic history, basic diseases, clinical symptoms, imaging findings, laboratory tests, and treatment measures were obtained from medical records.

### Grouping Methods

The degrees of COVID-19 infection were categorized into ordinary, severe, and critical based on *Diagnosis and Treatment Protocol for Novel Coronavirus Pneumonia (Trial Version 7)* (10). In brief, ordinary or mild patients exhibited mild clinical symptoms with or without imaging changes. Adult patients with severe type were characterized by at least one of the following symptoms: respiratory frequency ≥30/min, blood oxygen saturation at rest ≤93%, PaO_2_/FiO_2_ ratio <300 mmHg and lung infiltrates >50% within 24–48 h. Critical cases were those exhibiting respiratory failure with mechanical ventilation, septic shock and/or multiple organ dysfunction/failure, and they needed ICU monitoring. With the purpose of optimizing this study design, ordinary, and mild types were combined into the ordinary type in this study.

At the end of the study, each group was divided into smaller subgroups according to age, hypertension, highest temperature, chest tightness, glucocorticoid therapy, and hypoxemia to further analyze potential confounding factors.

### Statistical Analysis

SPSS (version 22.0) was used to perform statistical analyses. Continuous data with normal distribution were expressed as means ± standard deviation (SD) and were analyzed by analysis of variance (ANOVA). Continuous data with non-normal distribution medians were expressed as the interquartile range (P25-P75) and were analyzed by non-parametric test. Categorical data were expressed as numbers (%) and were compared by Chi-squared test. The Mann-Kendal test was used to test the trend of each liver function index with hospitalization time. When the sample size was insufficient, the Fisher exact test was adopted. A *p* < 0.05 was considered to indicate statistical significance.

### Study Approval

This study was approved by the Ethics Committee of the Huangshi Hospital of Traditional Chinese Medicine in Hubei Province (HSZYPJ-2020-021-01) in compliance with the principles of the Declaration of Helsinki and according to Good Clinical Practice guidelines. Written informed consent was waived due to the rapid emergence of this infectious disease.

## Results

### Demographic and Clinical Characteristics of COVID-19 Patients in Different Groups

As shown in Table 1, the average age in the critical group (71.9 ± 11.6) was significantly older than that in the ordinary (49.3 ± 14.1) and severe groups(61.4 ± 13.9). The frequency of patients with hypertension (19.4%) and cardiovascular complications (12.9%) in the critical group was higher than that in the ordinary (6.2%, 0.9%) and severe groups (17.4%, 4.3%).

**Table 1:** Demographics and baseline characteristics of patients infected with SARS-CoV-2.

**Characteristics**	**All patients**	**Disease severity**			***P*-value**
	**(*n* = 288)**				
		**Ordinary** **(*n* = 211)**	**Severe** **(*n* = 46)**	**Critical** **(*n* = 31)**	
Age (mean ±*SD*), yr	53.7 ± 15.8	49.3 ± 14.1	61.4 ± 13.9	71.9 ± 11.6	<0.001
**Age groups, No. (%)**					<0.001
<50 yr	112 (38.9)	103 (48.7)	8 (17.5)	1 (3.2)	
50–59 yr	72 (25.0)	59 (28.0)	10 (21.7)	3 (9.7)	
60–69 yr	59 (20.5)	36 (17.1)	14 (30.4)	9 (29.0)	
>70 yr	45 (15.6)	13 (6.2)	14 (30.4)	18 (58.1)	
**Sex, No. (%)**					0.679
Female	141 (49.0)	106 (50.2)	22 (47.8)	13 (41.9)	
Male	147 (51.0)	105 (49.8)	24 (52.2)	18 (58.1)	
Exposure history^†^, No. (%)	107 (37.2)	83 (39.3)	13 (28.3)	11 (35.5)	0.148
**Coexisting disorders, No. (%)**					
Hypertension	27 (9.4)	13 (6.2)	8 (17.4)	6 (19.4)	0.006^*^
Diabetes	24 (8.3)	14 (6.6)	6 (13.0)	4 (12.9)	0.187^*^
Cardiovascular disease	8 (2.8)	2 (0.9)	2 (4.3)	4 (12.9)	0.002^*^
Malignancy	3 (1.0)	1 (0.5)	1 (2.2)	1 (3.2)	0.175^*^
Chronic kidney disease	3 (1.0)	1 (0.5)	2 (4.3)	0	0.099^*^
Incubation (mean ±*SD*)^‡^, day	5.8 ± 4.1	5.5 ± 4.2	7.0 ± 3.2	6.4 ± 4.6	0.580

* *P-value of Fisher's exact test between two groups, T < 1 or 2 cells (25.0%) have T < 5*.

† *Represents a clear history of contact with infected patients*.

‡ *Represents days from possible contact with an infected person to illness onset*.

As shown in Table 2, the proportion of severe (47.8%) and critical (54.8%) patients with chest tightness at admission were significantly higher than that of ordinary patients (17.5%). High fever (more than 39°C) was more common in the severe group. During hospitalization, 21.2% of patients developed hypoxemia, of which that of severe patients (65.2%) and critical patients (51.6%) was significantly higher than that of ordinary patients (7.1%), and more severe patients (82.2%) and critical patients (80.8%) received glucocorticoid therapy compared with ordinary patients (16.5%). After standardized treatment, the averaged days of nucleic acid turning negative and CT symptoms disappear had significant differences among the three groups. In the critical group, only one patient achieved negative nucleic acid and CT symptoms disappeared before the study deadline. A total of 23 patients died, and the mortality of the critical group was significantly higher than that of the other two groups.

**Table 2:** Clinical characteristics and outcomes of patients infected with SARS-CoV-2.

**Characteristics**	**All patients**	**Disease severity**			***P-*value**
	**(*n* = 288)**				
		**Ordinary (*n* = 211)**	**Severe (*n* = 46)**	**Critical (*n* = 31)**	
**Signs and symptoms at admission, No. (%)**					
Fever	215 (74.7)	159 (75.4)	37 (80.4)	19 (61.3)	0.150
Dry cough	134 (46.5)	97 (46.0)	27 (58.7)	10 (32.3)	0.070
Fatigue	44 (15.3)	29 (13.7)	12 (26.1)	3 (9.7)	0.071
Chest tightness	76 (26.4)	37 (17.5)	22 (47.8)	17 (54.8)	<0.001
Expectoration	14 (4.9)	7 (3.3)	7 (15.2)	0	0.005^*^
Diarrhea	22 (7.6)	15 (7.1)	4 (8.7)	3 (9.7)	0.814^*^
Pharyngalgia	5 (1.7)	3 (1.4)	2 (4.3)	0	0.226^*^
Anorexia	27 (9.4)	16 (7.6)	9 (19.6)	2 (6.5)	0.050^*^
Dizzy	13 (4.5)	11 (5.2)	2 (4.3)	0 (0)	0.570^*^
**Treatments and outcomes**					
Glucocorticoid therapy, No. (%)	90 (34.0)	32 (16.5)	37 (82.2)	21 (80.8)	<0.001
**Highest temperature**, **°C**					0.005
<37.3	72 (25.0)	51 (24.2)	10 (21.7)	11 (35.5)	
37.3–37.9	70 (24.3)	64 (30.3)	4 (8.7)	2 (6.5)	
38.0–38.9	80 (27.8)	52 (24.6)	18 (39.2)	10 (32.3)	
≥39.0	66 (22.9)	44 (20.9)	14 (30.4)	8 (25.7)	
Hypoxemia, No. (%)	61 (21.2)	15 (7.1)	30 (65.2)	16 (51.6)	<0.001
Fever days ^§^	9.0 (7.0, 11.0)	8.0 (6.0, 11.0)	10.0 (7.0, 11.0)	11.0 (7.0, 14.8)	0.194
Nucleic acid turning negative days^§^	17.0 (13.0, 21.0)	16.0 (13.0, 20.0)	21.0 (17.0, 27.0)	14.0^†^	<0.001
CT symptoms disappear days^§^	14.0 (10.0, 19.0)	13.0 (10.0, 18.0)	20.0 (15.0, 26.0)	20.0^†^	<0.001
Inpatient days^‡^^,^ ^§^	18.0 (15.0, 23.0)	18.0 (15.0, 22.0)	23.0 (20.0,29.0)	18.0 (11.0, 26.0)	<0.001
Death, No. (%)	23 (8.0)	1 (0.5)	1 (2.2)	21 (67.7)	<0.001

* *P-value of Fisher's exact test between two groups, T < 1 or 2 cells (25.0%) have T < 5*.

† *Only one critical patient reported nucleic acid turning positive days and CT symptoms disappear days*.

‡ *Some patients have not been discharged, especially critical patients*.

§ *Non-normal distribution data expressed as median (Q1, Q3)*.

### Dynamic Changes in Liver Function Indexes in Different Groups of COVID-19 Patients During Hospitalization

**Figure 1:**
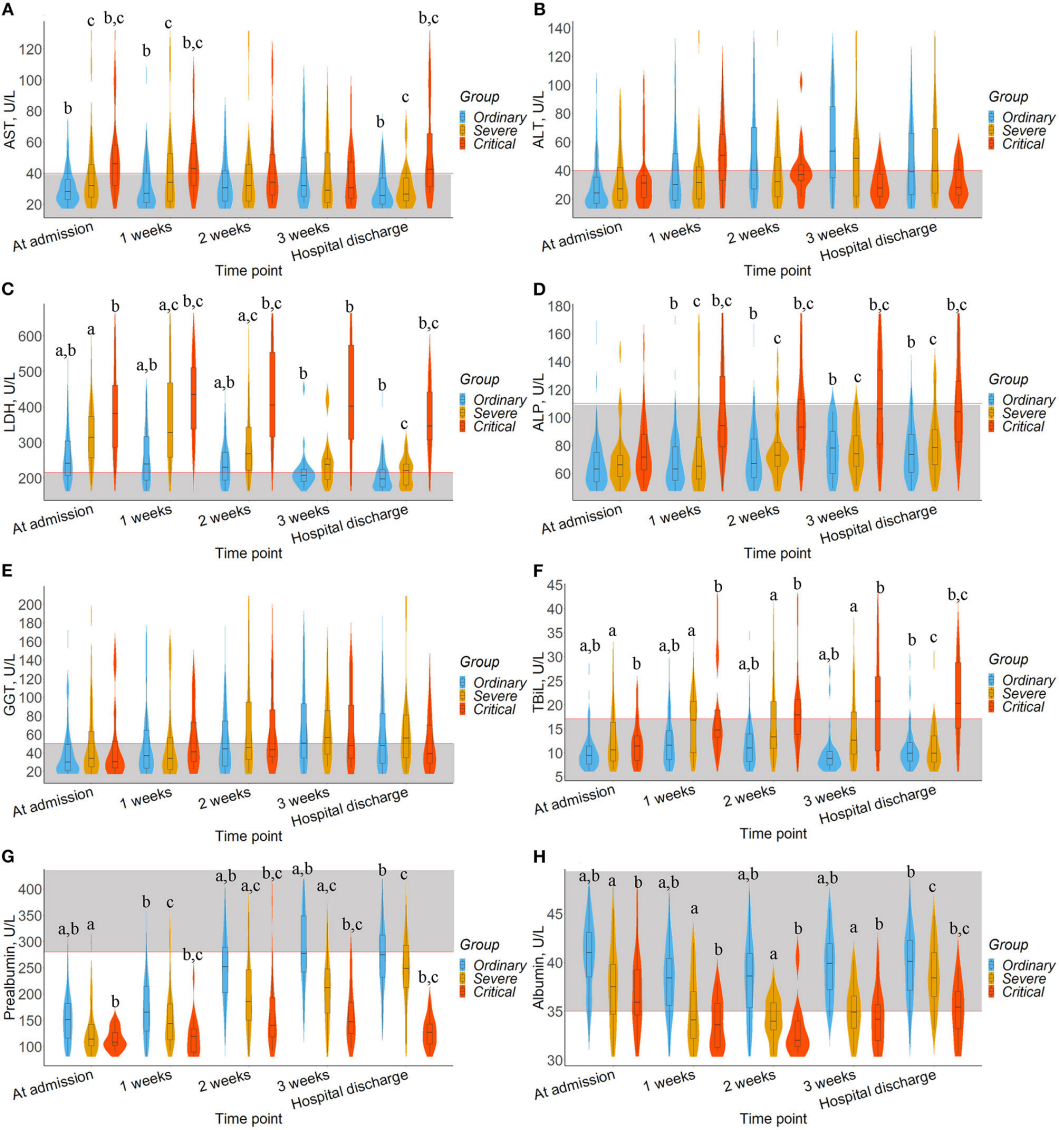
Timeline charts illustrate dynamic changes of liver biomarkers. The box in the violin plot represents the median and quartile, the extension from the thin black line represents the 95% confidence interval, and the width of the violin plot represents the sample size at this level. In addition, the normal range was defined and drawn as gray area. ^a^ represents the significant difference between the ordinary and severe groups after *Bonferroni* correction. ^b^ represents the significant difference between ordinary and critical group after *Bonferroni* correction. ^c^ represents the significant difference between severe and critical group after *Bonferroni* correction. **(A)** AST. **(B)** ALT. **(C)** LDH. **(D)** ALP. **(E)** GGT. **(F)** TBiL. **(G)** Prealbumin. **(H)** Albumin.

To observe the dynamic impact of SARS-CoV-2 infection and clinical treatment on the liver function of patients, a violin plot was used to show the results of AST, ALT, GGT, LDH, ALP, TBiL, prealbumin, and albumin at different times after admission, as shown in Figure 1. The trend of each liver biomarker index with hospitalization time according to the Mann-Kendal test showed in Supplementary Table 1.

The median levels of AST, TBiL, and ALP in ordinary and severe patients fluctuated within the normal range (Figures 1A,D,F). Moreover, the median levels of ALP in severe patients (*Z* = 2.021, *P* = 0.043) and the median levels of TBiL in critical patients (*Z* = 2.205, *P* = 0.027) had a significant upward trend. As shown in Figures 1B,E, ALT and GGT levels among the three groups were fluctuated around the normal range and showed an upward trend (*Z* > 0, *P* > 0.05), except for ALT median levels in critical patients. Differently, LDH level in critical patients fluctuated above the normal value (Figure 1C), higher than that of the ordinary and severe groups (*P* < 0.05) and showed a significantly downward trend in ordinary and severe patients (*Z* = −2.205, *P* = 0.028). We also observed that prealbumin levels among the three groups fluctuated below the normal value, showing an upward trend especially in the severe group (*Z* = 2.205, *P* = 0.027), and the critical group was lower than that in the other two groups (Figure 1G, *P* < 0.05). Albumin levels in the ordinary group fluctuated within the normal range, higher than the severe and critical groups (Figure 1H, *P* < 0.05), and their albumin level fluctuated around the normal.

### Changes From Baseline in Liver Biomarkers Among Different Groups of COVID-19 Patients at Discharge

As shown in Table 3, the baseline levels of AST and LDH in the critical group [(46.0, P25–P75:32.0–59.3), (390, P25–P75:290–516), respectively] were significantly higher than that in ordinary group [(28.0, P25–P75:23.0–38.0), (326, P25–P75:195–293), respectively], whereas prealbumin and albumin levels showed opposite trends. Also, ALP levels were increased than their baseline among three groups (8.0, 12.0, 25.0, respectively), and the change of the critical group was significantly higher than that of the ordinary group (*P* < 0.05). Furthermore, there were differences in the baseline levels of TBiL among three groups (*P* = 0.016), but no difference was found between the two groups after Bonferroni correction.

**Table 3:** Summary of changes from baseline in liver biomarkers at discharge.

**Characteristics**	**All patients (*n* = 288)**	**Disease severity**			*P-*value^*^
		**Ordinary (*n* = 211)**	**Severe (*n* = 46)**	**Critical (*n* = 31)**	
AST (U/L), *n*	221	160	41	20	
Baseline	31.0 (24.0, 43.0)	28.0 (23.0, 38.0)^‡^	32.0 (24.0, 54.5)	46.0 (32.0, 59.3)^‡^	<0.001
Change from baseline	−4.0 (−14.0, 4.0)	−4.5 (−12.8, 2.0)	−9.0 (−24.0, 1.5)^§^	6.0 (−11.3, 72.0)^§^	0.013
ALT (U/L), *n*	221	160	41	20	
Baseline	22.0 (15.0, 36.0)	21.5 (14.3, 35.8)	25.0 (15.0, 45.0)	26.5 (16.0, 35.8)	0.625
Change from baseline	9.0 (−0.5, 33.5)	9.5 (1.0, 35.5)	4.0 (−6.0, 37.5)	5.5 (−4.0, 19.8)	0.335
LDH (U/L), *n*	158	122	20	16	
Baseline	260 (203, 320)	326 (195, 293)^†^^,^^‡^	350 (269, 396)^†^	390 (290, 516)^‡^	<0.001
Change from baseline	−47 (−10, −6.8)	−46 (−95, −11.5)^†^^,^^‡^	−129(−177, −49)^†^^,^ ^§^	166 (−33, 397)^‡^^,^ ^§^	<0.001
ALP (U/L), *n*	200	147	36	17	
Baseline	60.0 (51.0, 73.0)	59.0 (51.0, 72.0)	61.0 (48.3, 72.0)	69.0 (57.5, 94.0)	0.100
Change from baseline	9.0 (−1.0, 23.8)	8.0 (−3.0, 20.0)^‡^	12.0 (1.3, 26.5)	25.0 (9.5, 83.0)^‡^	0.007
GGT (U/L), *n*	200	147	36	17	
Baseline	27.0 (19.3, 52.8)	26.0 (18.0, 49.0)	34.0 (23.3, 63.5)	25.0 (21.5, 83.5)	0.172
Change from baseline	5.0 (−1.8, 26.8)	4.0 (−2.0, 23.0)	15.5 (0, 30.0)	4.0 (−34.0, 38.5)	0.148
TBiL (μmol/L), *n*	200	147	36	17	
Baseline	9.3 (7.3, 11.8)	9.0 (7.1, 11.3)	10.1 (8.0, 15.4)	11.6 (8.2, 14.0)	0.016
Change from baseline	0.4 (−1.9, 3.5)	0.2 (−1.7, 2.8)^‡^	−1.3 (−2.9, 1.5)^§^	15.4 (4.8, 26.3)^‡^^,^ ^§^	<0.001
Prealbumin (mg/L), *n*	187	140	31	16	
Baseline	134 (99, 172)	145 (109, 180)^†^^,^^‡^	99 (73, 132)^†^	103 (61, 122)^‡^	<0.001
Change from baseline	124 (64, 179)	126 (74, 189)^‡^	146 (94, 177)^§^	7 (−41, 48)^‡^^,^ ^§^	<0.001
Albumin (g/L), *n*	200	147	36	17	
Baseline	39.9 (36.1, 42.6)	40.8 (37.2, 43.3)^†^^,^^‡^	37.5 (33.7, 39.7)^†^	35.6 (31.9, 39.3)^‡^	<0.001
Change from baseline	−0.6 (−3.9, 2.6)	−0.6 (−3.7, 1.8)	0.6 (−1.9, 4.2)^§^	−5.7 (−10.6, 4.0)^§^	0.022

* *Represents the P-values comparing different groups from the Kruskal-Wallis test*.

† *Represents the significant difference between the ordinary and severe group after Bonferroni correction*.

‡ *Represents the significant difference between the ordinary and critical group after Bonferroni correction*.

§ *Represents the significant difference between the severe and critical group after Bonferroni correction*.

*AST, aspartate aminotransferase; ALT, alanine aminotransferase; GGT, gamma-glutamyl transpeptidase; ALP, alkaline phosphatase; TBiL, total bilirubin; LDH, lactate dehydrogenase*.

At discharge, AST and LDH levels were decreased than their baseline in ordinary (−4.5, −46, respectively) and severe groups (−9.0, −129, respectively), while increased in critical group (6.0, 166, respectively), and the change of the critical group was significantly higher than that of the severe group (*P* < 0.05). In contrast to albumin, TBiL levels were increased in ordinary and critical groups (0.2, 15.4, respectively), while decreased in the severe group (−1.3). Prealbumin levels were increased among the ordinary, severe and critical groups (126, 146, 7, respectively).

For exploring the affecting factors of liver function in critical cases, we carried out a stratified analysis. We summarized that the factors affecting liver function (AST, ALP, and LDH) in critical patients included highest temperature ≥38.0°C, age ≥60 and symptom of hypoxemia (Supplementary Figures 1, 2, 4, 9, 11, 12). No significant associations between the variables (age, hypertension, highest temperature, chest tightness, glucocorticoid therapy, and hypoxemia) and the changed TBiL and prealbumin were observed (Supplementary Figures 5–8).

## Discussion

As of April 17, 2020, a total of 2,074,529 COVID-19 cases have been confirmed, with 139,378 deaths (11), and the epidemic is still expanding. In this retrospective study, the critical group had an average age of 71.9, 87.1% (27/31) were over 60.0, and it showed the highest fatality rate 67.7% (21/31). In view of the elderly patients often combined with chronic basic diseases and weakened immunity, their condition was more likely to deteriorate. Recent studies found that patients with a longer time from onset to admission are more likely to develop hepatic impairment (12), and pathological evidence also showed moderate microvascular steatosis and mild lobular inflammation in the liver tissue of patients with COVID-19 (13), indicating that COVID-19 infection can lead to liver injury in some patients. Moreover, Xie et al. found the increase of ALT or AST was also observed in nearly one-third of patients in non-ICU group (14). To further explore the effects of COVID-19 on liver function, we conducted a mono-centric study in the early phase of the pandemic to analyze the changes of serological hepatic biomarkers in COVID-19 patients during hospitalization and at discharge.

During hospitalization, we found LDH was increased in all three groups, especially exceeding the normal range in the critical group. After treatment, AST and LDH levels in ordinary and severe cases gradually tended to be normal, but it should be noted that the levels of LDH in critical cases were increased until discharge. There are various factors contributing to the elevation of LDH. Chen et al. (6) found that COVID-19 patients with cardiovascular events are more likely to have heart injury and heart failure, illuminating that the increase of LDH may also be related to heart function damage. A case-control study found that a high level of LDH was an independent factor associated with 1-month mortality in older COVID-19 inpatients (15). Moreover, a meta-analysis indicated that the abnormal changes of serological examination results, such as LDH, are related to multiple organ dysfunction and its severity (16). Increased liver function indicator levels, such as ALT, AST, ALP, and TBiL, were involved in the increased mortality risk of COVID-19 (17). However, in our study, the levels of raised ALT and AST were limited, which was consistent with the previous study (18). These findings showed the correlation between liver injury and the prognosis of the disease and the monitoring of LDH in COVID-19 patients, especially in critical patients should be paid enough attention.

At present, few researchers have reported a significant increase in serum ALP levels in COVID-19. The current study found that the median levels of ALP and GGT among the three groups shown an upward trend, especially the increasing trend of ALP median levels in severe patients was significant. Apart from the effects of age, elevated ALP may imply the injury of the bile duct. It has been fully confirmed that the co-expression of angiotensin-converting enzyme 2 (ACE2) (19, 20) and transmembrane protease serine 2 (TMPRSS2) (21, 22) is necessary for SARS-CoV-2 to enter the cells. Trophoblast cell surface antigen 2 (TROP2) protein is expressed in putative bipotent liver epithelial progenitors as well as biliary cells (23). Moreover, a recent scRNA-seq analysis reported that adult human liver TROP2+ progenitors co-express ACE2 and TMPRSS2 (24), indicating that the liver could be a potential target of SARS-CoV-2. However, previous results of sequencing showed that the expression of ACE2 in hepatocytes was very low, while the expression of ACE2 in bile duct epithelial cells was 20 times higher than that in hepatocytes (15, 25). Given that ACE2 is the crucial factor (26), SARS-hepatic inflammation may be more likely to induce bile duct epithelial cell damage than direct liver damage. Combined with our detection of markedly increased TBiL in the critical group, it is speculated that SARS-CoV-2 could induce the injury of bile duct cells and then bring certain damage to liver function.

Albumin and prealbumin are valuable indicators that are capable of predicting poor body status and clinical prognosis of numerous diseases (27–29). In our study, prealbumin and albumin levels were increased among ordinary and severe groups at discharge, but the median of prealbumin was still below the limit of the normal range. Liver dysfunction can lead to hypoalbuminemia. Hypoalbuminemia and elevated AST levels were often observed in critical patients, and the correlation of albumin and AST levels with disease severity was also found (30). Therefore, it can be considered that there existed substantial hepatic dysfunction. Albumin levels in both severe and critical groups were <40.0 g /L. Also, the level of the ordinary group was higher than that of the other two groups. And in all three groups, the levels decreased first and then increased during hospitalization, which indicated an improving trend of status as well as suggested that prealbumin and albumin might be related to the condition of patients.

Clinically, COVID-19 patients with fever usually received antipyretic treatment during hospitalization. Most antipyretic drugs contain paracetamol, which has been recognized to cause serious liver injury or even induce liver failure. In addition, although there is no targeted antiviral treatment for COVID-19, many patients still take non-specific antiviral drugs such as lopinavir and ritonavir, which may have certain hepatotoxicity and induce liver injury.

We noted that critical cases had severe symptoms of chest tightness at admission, and the proportion of oxygen saturation deficiency in severe and critical cases was as high as 65.2 and 51.6%, respectively. Researchers have found oxygen deprivation, lipid accumulation, glycogen consumption and ATP depletion in hepatocytes can rapidly lead to hepatocyte death (31). With the increase of reactive oxygen species (ROS), ROS and its peroxides act as the second messengers, activate redox-sensitive transcription factors, further activate the release of a variety of pro-inflammatory factors and then lead to liver damage (32). This indicates that the hypoxic internal environment could be one of the secondary injury factors in COVID-19 patients.

This study still has some limitations. Firstly, most dead critical cases are lack autopsy reports, and the pathological changes in the liver cannot therefore be observed in detail. Secondly, it cannot be ruled out that some changes in liver function in critical cases could also be secondary to the dysfunction of other organs or sepsis, etc. Finally, for most of the variables, data were available only for some patients and not all, and the changes in most of the variables, though different across groups, were not very remarkable.

In summary, we observed that COVID-19 has caused changes in several liver biomarkers, which may be closely related to the severity of the disease. In this study, critical cases had a worse prognosis, with higher fatality and worse liver function, for which the changes of liver biomarkers should be closely monitored, especially LDH, ALP, GGT, TBiL, prealbumin, and albumin. This will help evaluate and predict disease prognosis and disease severity in COVID-19. In addition, hepatotoxic drugs should be used with great caution during clinical treatment, and liver protection drugs could be applied appropriately when necessary.

## Data Availability Statement

The original contributions generated for the study are included in the article/Supplementary Material, further inquiries can be directed to the corresponding author/s.

## Ethics Statement

The studies involving human participants were reviewed and approved by Ethics Committee of the Huangshi Hospital of Traditional Chinese Medicine in Hubei Province (HSZYPJ-2020-021-01). Written informed consent for participation was not required for this study in accordance with the national legislation and the institutional requirements.

## Author Contributions

CZ conceived the study and assumed responsibility for the paper as a whole. HF and CZ provided statistical advice and analyzed the data. JC, AT, YL, and HY drafted the manuscript. ZJ, YY, LR, PH, MY, NC, and JL collected the data. All of the authors contributed substantially to its revision, read, and approved the final manuscript.

## Conflict of Interest

The authors declare that the research was conducted in the absence of any commercial or financial relationships that could be construed as a potential conflict of interest.
