# Supplementary material for: Comparison of Liver Biomarkers in 288 COVID-19 Patients: A Mono-Centric Study in the Early Phase of Pandemic
Source: Front Med (Lausanne). 2021 Jan 15;7:584888. doi: 10.3389/fmed.2020.584888 (PMC7843802; doi:10.3389/fmed.2020.584888)
Supplement: Supplementary file 1 [file Data_Sheet_1.docx]

**Supplementary Table 1. Trend of each liver function index with hospitalization time according to** **Mann-Kendal test**

| Characteristics | Disease Severity | | |
| --- | --- | --- | --- |
|  | Ordinary n= (211) | Severe (n= 46) | Critical (n=31) |
| AST (U/L), n |  |  |  |
| Z | 0 | -0.735 | 0 |
| *P* value | 1 | 0.462 | 1 |
| ALT (U/L), n |  |  |  |
| Z | 1.225 | 1.516 | 0 |
| *P* value | 0.221 | 0.130 | 1 |
| LDH (U/L), n |  |  |  |
| Z | -2.205 | -1.715 | 0.735 |
| *P* value | **0.028** | 0.086 | 0.462 |
| ALP (U/L), n |  |  |  |
| Z | 1.715 | 2.021 | 1.715 |
| *P* value | 0.086 | **0.043** | 0.086 |
| GGT (U/L), n |  |  |  |
| Z | 1.225 | 1.715 | 0.735 |
| *P* value | 0.221 | 0.086 | 0.462 |
| TBiL (μmol/L), n |  |  |  |
| Z | -0.225 | -0.735 | 2.205 |
| *P* value | 0.807 | 0.462 | **0.027** |
| Prealbumin (mg/L), n |  |  |  |
| Z | 1.715 | 2.205 | 0.735 |
| *P* value | 0.086 | **0.027** | 0.462 |
| Albumin (g/L), n |  |  |  |
| Z | 0.245 | 0.245 | -0.735 |
| *P* value | 0.807 | 0.806 | 0.462 |

**Supplementary Figures**


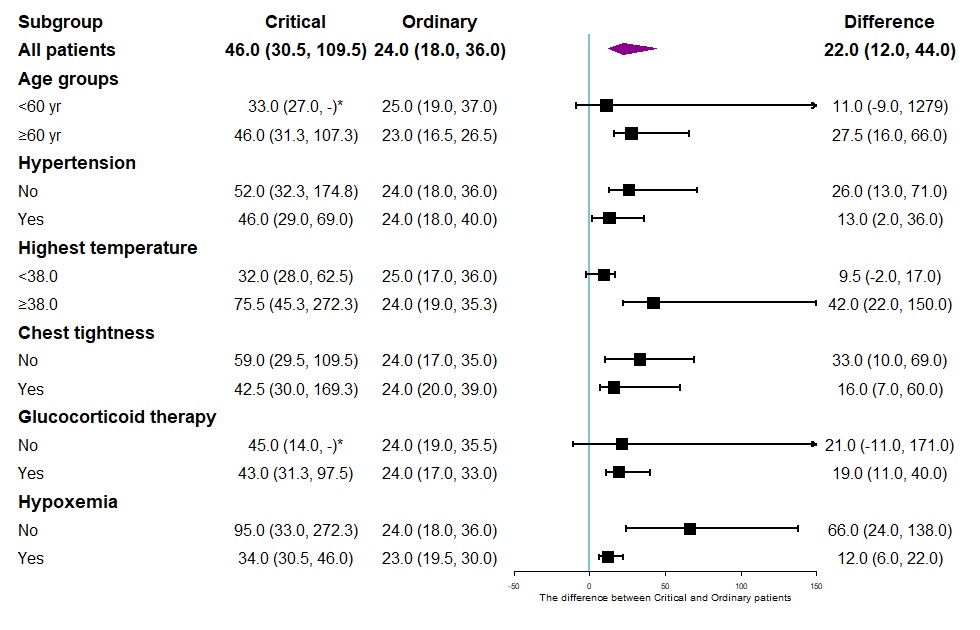


Figure S1. Differences in the AST levels at discharge between critical cases and ordinary cases.

* sample size is less than 5.


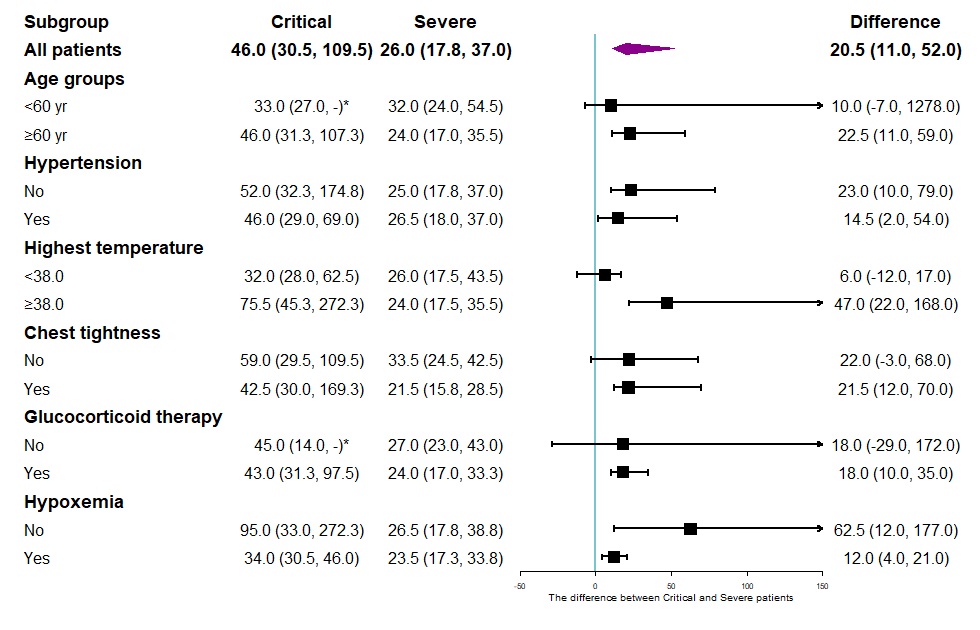


Figure S2. Differences in the AST levels at discharge between critical cases and severe cases.

* sample size is less than 5.


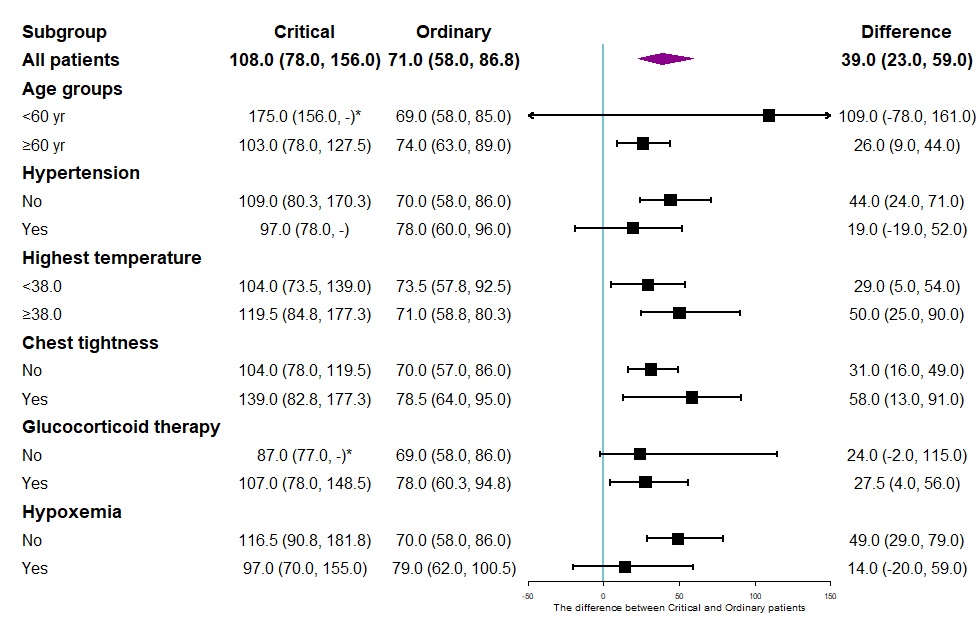


Figure S3. Differences in the ALP levels at discharge between critical cases and ordinary cases.

* sample size is less than 5.


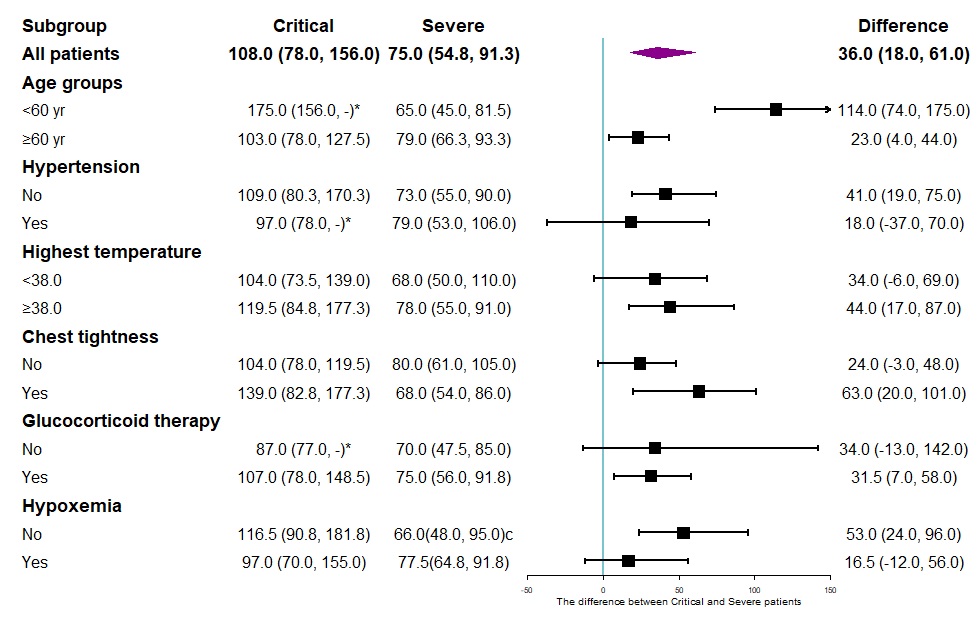


Figure S4. Differences in the ALP levels at discharge between critical cases and severe cases.

* sample size is less than 5.


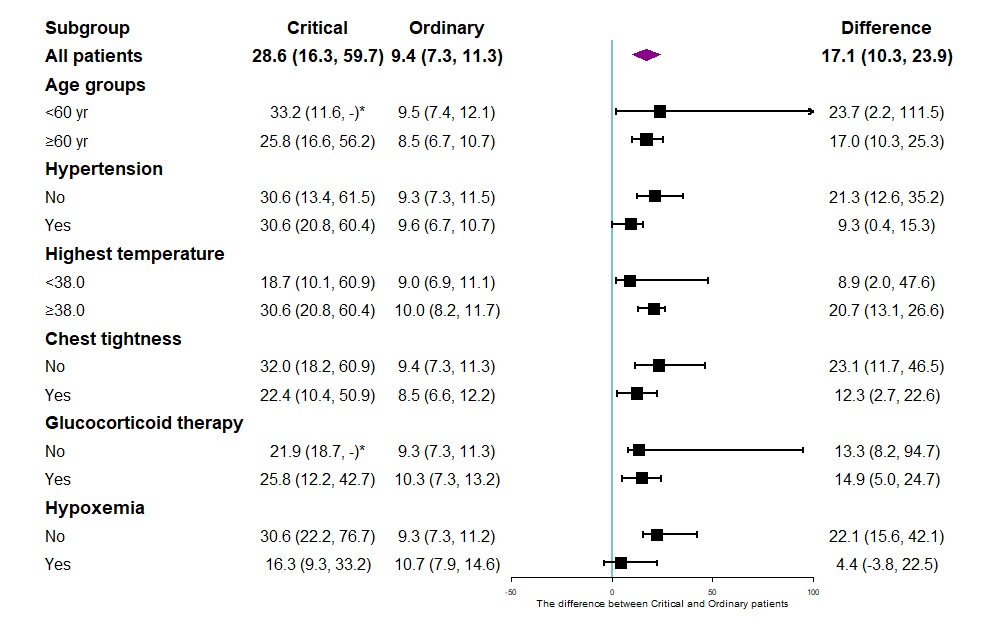


Figure S5. Differences in the TBiL levels at discharge between critical cases and ordinary cases.

* sample size is less than 5.


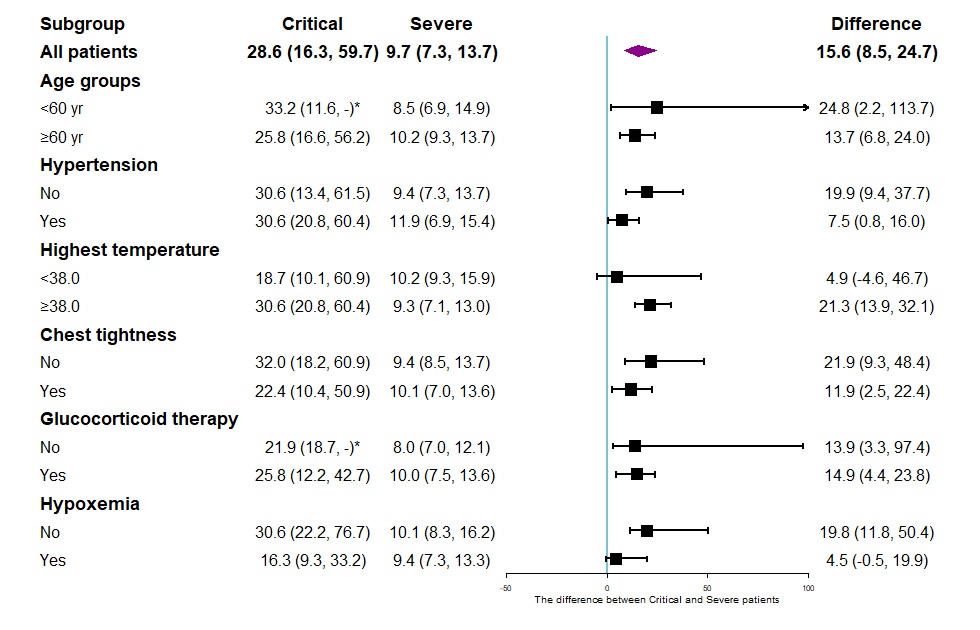


Figure S6. Differences in the TBiL levels at discharge between critical cases and severe cases.

* sample size is less than 5.


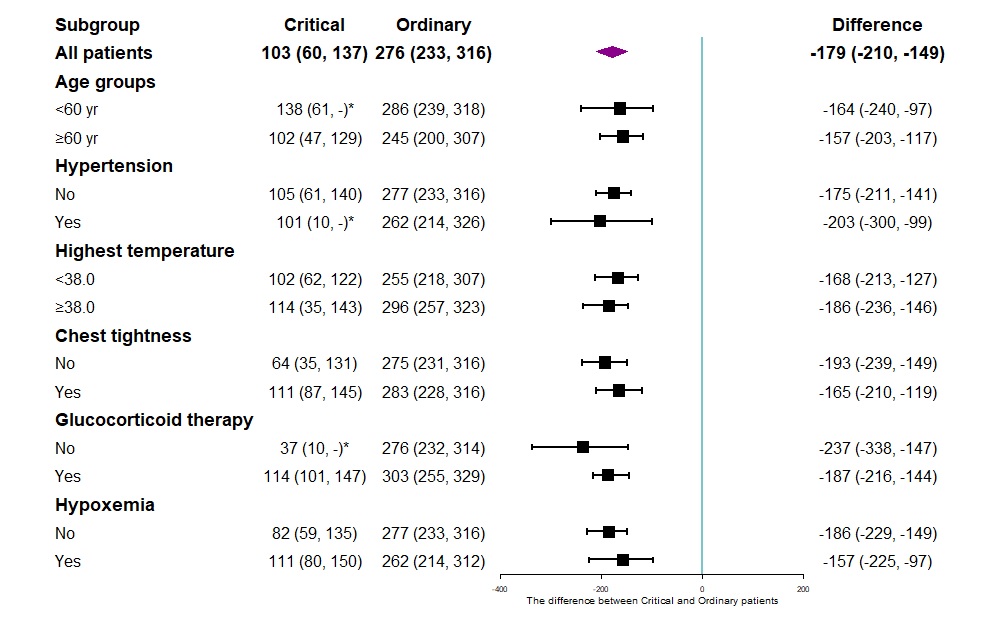


Figure S7. Differences in the prealbumin levels at discharge between critical cases and ordinary cases.

* sample size is less than 5.


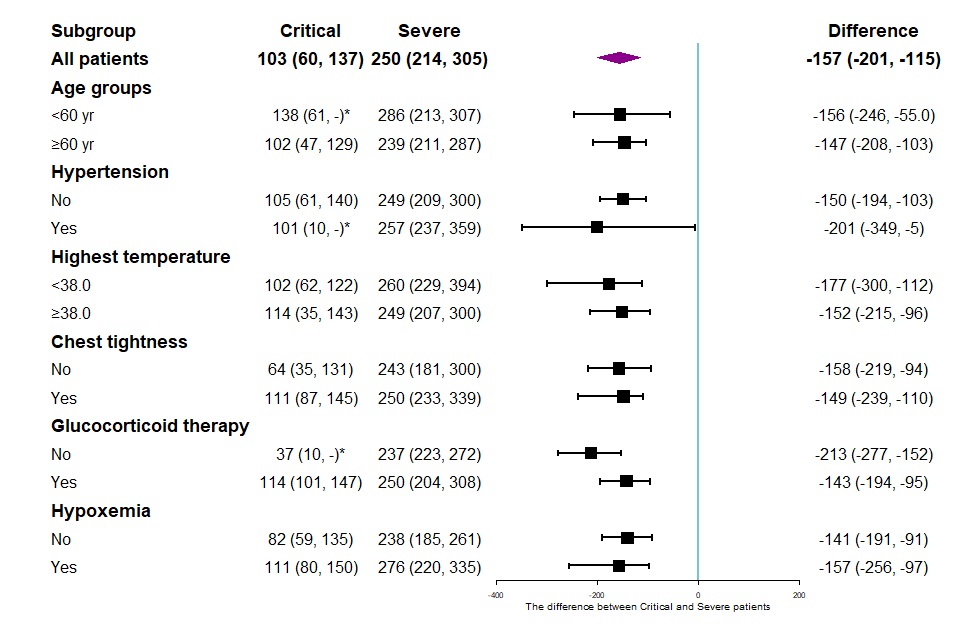


Figure S8. Differences in the prealbumin levels at discharge between critical cases and severe cases.

* sample size is less than 5.


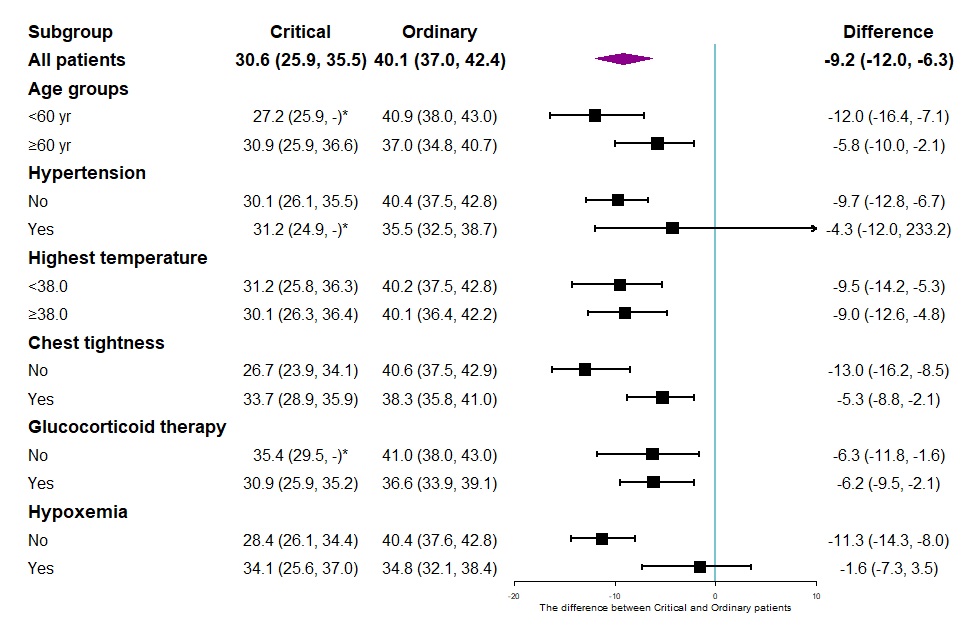


Figure S9. Differences in the albumin levels at discharge between critical cases and ordinary cases.

* sample size is less than 5.


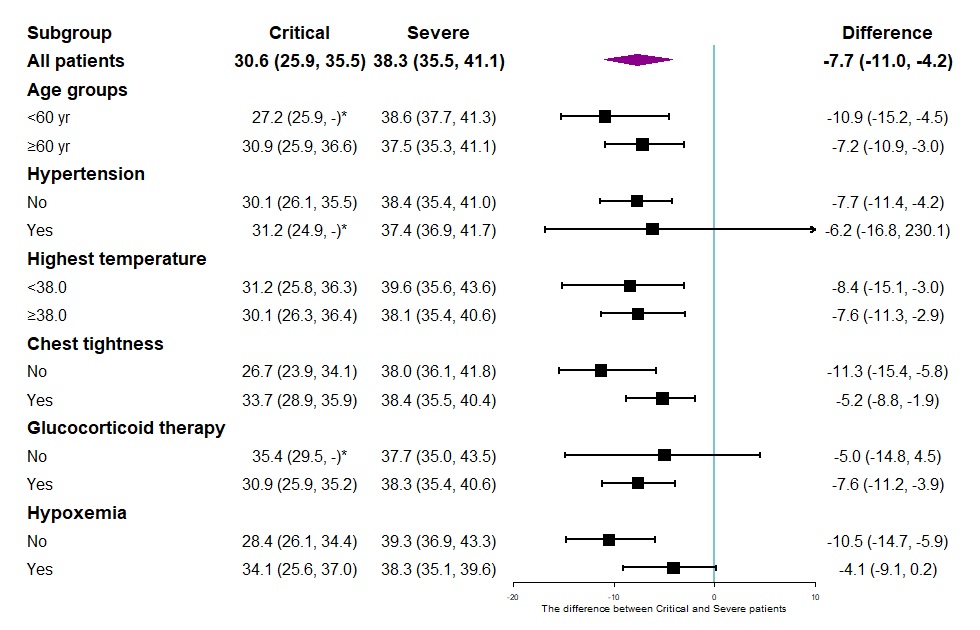


Figure S10. Differences in the albumin levels at discharge between critical cases and severe cases.

* sample size is less than 5.


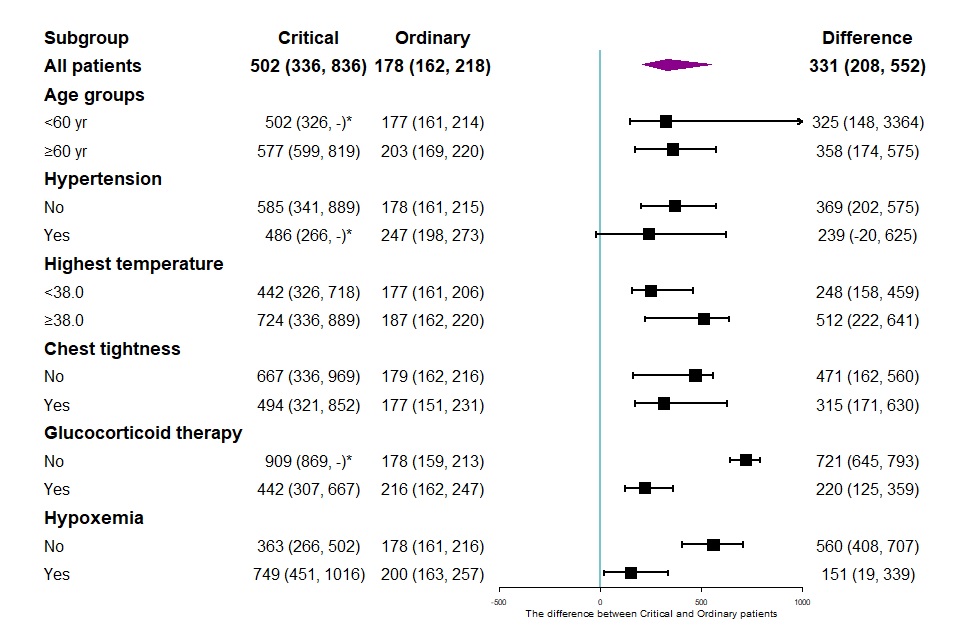


Figure S11. Differences in the LDH levels at discharge between critical cases and ordinary cases.

* sample size is less than 5.


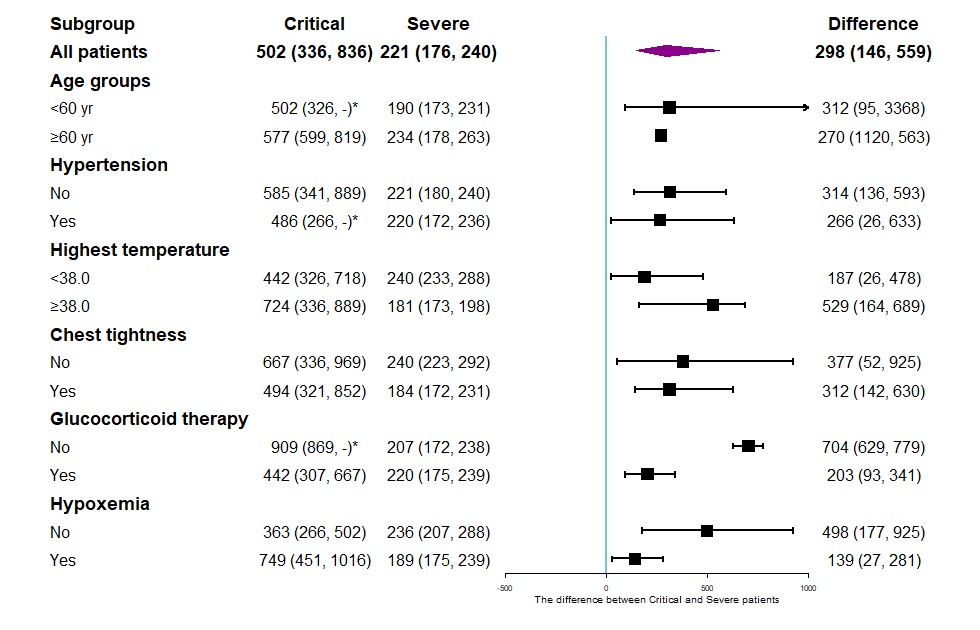


Figure S12. Differences in the LDH levels at discharge between critical cases and severe cases.

* sample size is less than 5.
